# CHANGE IN SOCIAL PARTICIPATION OVER TIME AMONG PERSONS AGES 45–64 AGING WITH LONG-TERM DISABILITY

**DOI:** 10.1093/geroni/igae098.2710

**Published:** 2024-12-31

**Authors:** Michelle Putnam, Susan Stark, Kerri Morgan, Holly Hollingsworth, Melissa Krauss

**Affiliations:** Simmons University, Boston, Massachusetts, United States; Washington University in St. Louis, St. Louis, Missouri, United States; Washington University School of Medicine, St. Louis, Missouri, United States; Washington University School of Medicine, St. Louis, Missouri, United States; Washington University School of Medicine, St. Louis, Missouri, United States

## Abstract

Change in social participation over time among mid-life adults with disabilities as they grow older is under-studied. Existing research indicates that social participation among persons with disabilities, younger and older, is positively associated with health and well-being outcomes including mental health and life satisfaction. Despite this knowledge, few studies have explored if and how social participation changes year to year, and how symptoms of aging with disability including pain, fatigue, and depression may influence those changes. We undertook a cohort survey (2019-2021) with a sample of adults aged 45-64 (N=323) with self-reported mobility disability, collecting data over three annual time points, in order to better understand if we could see annual change in social participation that could help inform early interventions to support and sustain social participation. Our results found that T-scores from PROMIS measures of physical function, depression, and pain changed little over time, but level of fatigue did significantly change. PROMIS T-scores of satisfaction with social roles and activities and did not change over time, but T-scores for ability to participate in social roles and activities increased over time. T-Scores for physical function, depression, fatigue, and pain were significantly associated with both participation outcomes. In qualitative data, most participants (63%) said their participation declined over the 3 years, attributing change to health, aging with disability symptoms, and the COVID-19 pandemic. Study limitations include potential effects of the COVID-19 pandemic on participation and disability/health. Findings suggest the need to consider aging with disability symptoms when evaluating participation interventions.

